# Improving the physician-patient relationship utilizing psychodynamic psychology: a primer for health professionals

**DOI:** 10.1080/21642850.2021.1914052

**Published:** 2021-04-17

**Authors:** Luis A. Centeno-Gándara

**Affiliations:** Department of Psychiatry, Faculty of Medicine and University Hospital ‘Dr. José Eleuterio González’, Autonomous University of Nuevo Leon, Monterrey, Mexico

**Keywords:** Countertransference, empathy, medical psychology, physician-patient relations, transference

## Abstract

**Background:**

The quality of the physician-patient relationship is associated with improved healthcare outcomes and patients’ complaints due to dissatisfaction. Factors that influence the quality of the physician-patient relationship include verbal communication, nonverbal communication, and clinical empathy. These factors have been studied from diverse theoretical approaches hindering their integration into a theoretical framework applicable in clinical practice and accessible for lay clinicians.

**Objective:**

The aim of this paper is to put forward a psychodynamic framework that includes the factors associated with a better quality of the physician-patient relationship and is applicable in clinical practice and accessible for lay clinicians.

**Theoretical discussion:**

Basic concepts necessary to comprehend transference and countertransference phenomena were outlined. Then, based on the concepts of transference and countertransference, a psychodynamic framework to understand and manage the physician-patient relationship was put forward.

**Conclusion:**

This is the first paper that presents a psychodynamic framework applicable in clinical practice and accessible to lay clinicians for understanding and managing the physician-patient relationship. Additionally, this work could serve as introductory material to Balint groups.

The physician-patient relationship is of paramount significance for the quality of patient's healthcare. It is the matrix in which clinical practice takes place. The quality of the physician-patient relationship has been empirically associated with improved healthcare outcomes such as weight loss, re-consultation rate, pain relief, asthma-related quality of life (Kelley, Kraft-Todd, Schapira, Kossowsky, & Riess, 2014), adherence to treatment (Arbuthnott & Sharpe, 2009; Haskard Zolnierek & DiMatteo, 2009), emotional health, fewer diagnostic tests and referrals, patient's satisfaction, improved treatment-related behavior, less aggressive medical care at the end of life, lower healthcare cost, better HbA1C levels, and improved patient well-being and quality of life (Riedl & Schüssler, 2017), among others. The quality of the physician-patient relationship also influences complaints and legal actions motivated by patients’ dissatisfaction instead of medical errors (Lussier & Richard, 2005).

The factors associated with a better quality of the physician-patient relationship include paying close attention to patients’ verbal communication to recognize their expectations and preferences, facilitating patients to verbalize their emotions, paying attention to and interpreting patients non-verbal communication, and the empathic ability of the clinician (Gómez & Aillach, 2013; Jani, Blane, & Mercer, 2012; Mast, 2007; Riedl & Schüssler, 2017; Squier, 1990). Because these factors have been studied from diverse, complex, and highly specialized empirical and theoretical approaches—spanning from neurophysiology to social approaches—, they have not been integrated into a theoretical framework applicable in clinical practice by lay clinicians (Gómez & Aillach, 2013).

Can psychodynamic psychology be used as a theoretical framework to understand and manage the physician-patient relationship? Yes. Psychodynamic psychology has long studied all the aforementioned factors associated with a better quality of the physician-patient relationship through its two signature concepts: transference and countertransference. Psychodynamic psychology refers to a conceptual framework that includes drive theory, ego psychology, object relations theory, self psychology, and attachment theory, among others.^1^ Unfortunately, most psychodynamic literature presupposes readers are knowledgeable in psychological and psychodynamic concepts and is inaccessible to lay clinicians.

In this this paper, I attempt to present a psychodynamic model of the physician-patient relationship for an audience with little familiarity with psychodynamic theory. First, I present basic concepts necessary to comprehend transference and countertransference phenomena. Then, based on the concepts of transference and countertransference, I attempt to put forward a psychodynamic framework to understand and manage the physician-patient relationship. Theoretically, following this framework would improve the quality of the physician-patient relationship, and thus improve healthcare outcomes.

## Basic concepts

### Cognition, affect, motivation, and behavior

All psychological phenomena can be classified into two categories: mental and behavioral phenomena. Mental phenomena are subjective; they can only be known by those who experience them because they are confined to the psyche of each individual and are not present in the observable physical world. Behavioral phenomena can be recognized by other individuals in addition to the person who executes the behavior because they are expressed in the observable physical world (Russell, 2016, pp. 47–64). The thought "I’m reading this article" is a mental phenomenon, since only the individual who thinks it can know it exists. The act of moving one's arms to turn the page in a book is a behavioral phenomenon because the person who turns the page as well as any other person who observes this with attention knows that the behavior exists in the observable physical world.

Mental phenomena can be further classified into three subcategories: cognition, affect, and motivation (Hilgard, 1980). The term cognition could be defined as the set of mental phenomena that correspond to thought and perception (Carter & Shieh, 2015). "I’m hungry" is a thought, whereas seeing a red colored glass in front of us is an instance of perception. Affect could be defined as the mental phenomenon that accompanies every human experience and defines it as agreeable or disagreeable (Segarra & Eguíluz, 2013; Russell, 2016, pp. 269–279). Love, hate, and anxiety, for example, are affective phenomena. Motivation is the psychological phenomenon that causes an individual to present a behavior (Brenner, 1974, pp. 15–30).

From a psychodynamic point of view, there are four types of motivations: the search for pleasure through perception, namely, libido; aggressive impulses; the desire to attach to others; and narcissistic needs, namely, the desire to reach one's maximum attainable development (Auchincloss, 2015, pp. 239–260). Let us look at examples in the patient-physician context. In medical practice, these motivations can be easily detected; for example, the diabetic patient who does not take his or her drugs could be motivated by a self-aggressive impulse. The somatizing patient who repeatedly goes to the emergency room to receive medical attention despite clinicians repeatedly telling him or her that there are no signs of physical disease could be behaving motivated by an impulse of dependence, and so on. The concept of motivation implies intentionality, meaning that all mental phenomena are directed to an object that is not the mental phenomenon itself (Russell, 2016, pp. 269–279). In clinical practice, these objects are the physicians.

Behavior refers to all body movements (Russell, 2016); it is the objective correlate of motivation (Eagle, 2012). Walking, frowning, and speaking are examples of behaviors.

### The unconscious

Consciousness could be defined as all the mental contents that are within the subjective experience of a person at a given moment. By deduction, the unconscious can be understood as the mental content that is not within the subjective experience of an individual at a given moment. For example, if one is asked, "How old are you?", a number emerges in the mind. This mental content, which corresponds to the number of years lived, is now conscious, but it had remained in the unconscious until a few seconds ago. Some unconscious mental contents, as our age, can be easily brought to consciousness, but there are other mental contents that are extremely difficult to bring to the conscious mind. Examples include the reasons why we love as we love or why we relate to others—including clinicians—as we do (Gabbard, 2014). All psychological phenomena can be conscious or unconscious.

### Self and object representations

Representations are cognitive phenomena, mental symbols that represent objects from the observable physical world (Auchincloss, 2015, pp. 269–292; Pitt, 2018). Imagine a pencil in your mind: 15 centimeters of length, yellow color, and hard texture. This is an instance of a representation.

A self representation is a mental representation that contains the positive and negative mental and body characteristics of an individual: our physical appearance; our needs and desires; our cognitive, affective, behavioral, and interpersonal traits; our ideal self, namely, the person we want to become; and moral beliefs (Jacobson, 1964). The self representation that a patient activates in a given moment can be inferred through the patient's verbal and nonverbal communication. For example, imagine that a clinician who just diagnosed one of their patients with breast cancer, asks the patient the following: "How are you feeling and what are you thinking after getting the diagnosis?" She says: "I’m very afraid. I never thought I would be in this situation, but I will face the disease and overcome it, I will not be defeated". Based on this response, it can be inferred that the self representation activated in the patient is that of a person surprised by the bad news, but someone who is brave and has the desire to fight against the disease. A contrasting self representation would be of someone who is defeated and hopeless.

An object representation corresponds to the mental phenomenon that represents an individual who is in the observable physical world. It is equivalent to the previously described self representation, but assigns characteristics to a person who is not the subject whose mind contains the representation. Let us return to the previous case and let us imagine that the patient who has now been diagnosed with breast cancer is asked what she thinks of the clinician who gave her the diagnosis. She says: "I think that you’re just doing your job and I thank you for informing me with the tactful way you did". We can infer that the object representation that the patient activates in this encounter with the clinician is that of a responsible, kind, and empathetic person.

### Internalized object relations

An internalized object relation is an affective–cognitive phenomenon that corresponds to a mental representation of an interpersonal relationship that is stored as memory—the interpersonal relationship is in the observable physical world, whereas the internalized object relation is in the mental world. Internalized object relations are composed of a self representation interacting with an object representation and the affect associated with this kind of interaction (Caligor, Kernberg, Clarkin, & Yeomans, 2018). Internalized object relations are established during the early development of individuals when we interact with our primary caregivers. Each one of the specific internalized object relations that have been stored in memory is called an object relation dyad (Yeomans, Clarkin, & Kernberg, 2015, pp. 149–212). A person contains a limited number of stored dyads, and based on these dyads, builds the knowledge of him or herself, of others, and later, of the world around him or her—at the beginning of life, the world of the newborn includes only him or herself and his or her primary caregiver, as time goes by and "hatching" occurs, his or her world expands to include other things, such as secondary caregivers and inanimate objects (Gilmore & Meersand, 2014).

At all times, a person has a specific object relation dyad activated to relate with others or the world that surrounds them. Even at this moment, for example, the reader has one active: Is it that of the demanding reader (self representation) who is frustrated (affect) by the poor expressive quality of the author (object representation), or is it that of the avid reader, satisfied with the text, who considers the author perspicacious?

Heretofore, I have presented the basic concepts necessary to comprehend transference and countertransference phenomena. Now, I am going to attempt to put forward a theoretical framework to understand and manage the physician-patient relationship utilizing psychodynamic psychology.

## Transference and countertransference in the physician-patient relationship

### Transference

Transference is the set of cognitions, affect, and motivations that the patient experiences about the physician (Hersh, Caligor, & Yeomans, 2016). The nature of the transference is determined by the token of the object relation dyad activated in the patient's mind that represents the interpersonal relationship formed with the clinician (Yeomans et al., 2015, pp. 149–212). Every transference can be classified, based on the affect that it includes, as positive or negative (Cabaniss, Cherry, Douglas, & Schwartz, 2017, pp. 234–253). Positive transferences include affects such as love, fondness, respect, and gratitude. Negative transferences include affects such as anger, hatred, distrust, disdain, contempt, and envy. Imagine a patient who has activated a dyad that contains a self representation of a sick person who needs to be taken care of interacting with an object representation of the physician, who tries his or her best to heal him or her, and therefore, the patient feels appreciation and gratitude. This is a classic example of a positive transference activated by a patient when interacting with their clinicians. Now imagine a patient who activated a dyad that includes a self representation of an ill person that requires help, but in relation to a representation of the physician as cold and without interest in helping him or her; therefore, the affect it contains is frustration and anger. This an example of a very prevalent negative transference in everyday clinical practice.

To diagnose the activated dyad in the transference, it is necessary for the physician to pay attention to three sources of information: the patient's verbal communication, non-verbal communication, and the countertransference of the practitioner. Verbal communication includes the information that the patient transmits through speech; non-verbal communication includes the nature of speech—tone, intensity, speed and fluctuations in these variables— and body language—facial expression, body posture and directed and non-goal-directed behavior—; and countertransference includes the affect that the clinician experiences when interacting with the patient (Yeomans et al., 2015, pp. 213–271; Hall, Horgan, & Murphy, 2019).

Each one of us must find the most effective personal way to diagnose an activated dyad using the aforementioned three sources of information. However, a simple and pragmatic method consists of diagnosing the patient's affect through non-verbal communication. Does the patient's facial expression correspond to what would be socially interpreted as an expression of positive or negative affect? Is he or she laughing or frowning? Is he or she very expressive or dour? What is the patient's posture? Is he or she straight and firmly seated against the back of the chair or sprawled in his seat? Does he or she play with their fingers showing anxiety or do they lie quietly on an object or a patient's body part? and so on. Once the activated affect has been diagnosed, the self and object representations involved must be diagnosed. These representations can be inferred from the patient's verbal communication, either directly when the patient speaks about him or herself or about the clinician with whom he or she interacts, or indirectly when the patient does not talk about him or herself or the physician, but of material that is not directly related to the physician-patient interaction (Joseph, 1985). In an earlier section of this paper titled "Self and object representations", examples of the inference of self and object representations through direct verbal communication have been presented. To illustrate the diagnosis of an activated dyad in the transference when the patient uses "indirect" verbal communication, imagine a patient who is due for follow-up for systemic arterial hypertension who presents in the clinic with high blood pressure. The practitioner asks routinely if he or she has taken their medication correctly, and the patient, rushing to answer, says yes; however, the clinician notices tension and discomfort in the patient's voice and facial expression—use of nonverbal communication—and decides to point it out by saying the following: "I noticed you felt uncomfortable when I asked if you had taken your medication, could it be that you have forgotten it sometimes and you are embarrassed to tell me?" The patient acknowledges it—we have the affect: shame—, and the physician then explores the motivation for not taking the medication. The patient answers, "The new drug that you prescribed was very expensive and I couldn't afford it. I’m sorry"—indirect verbal communication. This is an instance of indirect verbal communication because the patient does not directly describe the characteristics of the physician with whom they are interacting. From this comment, it can be inferred that the activated self representation corresponds to the misbehaved patient associated with a negative affect, that is, shame, coupled with an object representation of a critical clinician (and perhaps inconsiderate for prescribing expensive drugs).

In general, positive transferences strengthen the therapeutic alliance and do not require any intervention (Caligor, Kernberg, & Clarkin, 2007). If a clinician finds a negative transference in one of their patients, the clinician should express the active transference verbally to obtain confirmation from the patient that the hypothesis the physician has about the transference corresponds to the patient's experience. Once the transference hypothesis has been confirmed by the patient, the physician can request the patient for subjective and objective proofs, in favor and against, to support the veracity of his or her transference. With the information obtained through these interventions, the clinician can now reduce the intensity of the negative transference by declaring the reality of the physician-patient interaction, the manner in which the clinician experienced this interaction with the patient, and recognize his or her potential contribution to the biased perception of the patient. Finally, in the event that the clinician is aware that this transference has been activated in previous interactions with other physicians and that it has hindered the patient's treatment, the physician can point out this similarity with the objective of reducing the intensity of the same negative transference in the future (Kernberg, 2018). Let us continue with the example of the hypertense patient mentioned earlier: the physician, after having diagnosed the activated dyad in the transference, verbally expresses it to the patient to obtain confirmation: "I got the impression that at this moment, you feel as if you have misbehaved and you’re embarrassed by this and that I was being harsh or critical of you for not taking your medication. Is that so?" The patient answers affirmatively and then the clinician asks for evidence for or against this perception, resulting in the patient being unable to provide any. Finally, the clinician reduces the intensity of the negative transference by stating: "Let us work together to find a drug that's right for you. It is true that I didn't think that you would not be able to pay for this new drug", thus ending the intervention to reduce the intensity of the negative transference.

### Countertransference

Countertransference is the set of cognitions, affects, motivations, and behaviors that the physician experiences towards the patient (Hofsess & Tracey, 2010). Countertransference is a useful tool to infer the subjective experience of the patient, helping to better understand the affects and the self and object representations of the patient (Clarkin & Yeomans, 2013); its recognition decreases the risk of the clinician deviating from professional boundaries due to the strong countertransference reactions that some patients can induce.

Similar to how transference is classified, it is useful to classify countertransference based on the affect it includes as positive and negative countertransference. Intense positive and negative countertransferences carry a risk that the physician might reassure inappropriately—such as affirming a patient that there is no problem because they have not taken their medication, when there actually is, or attack the patient, respectively (Bateman & Holmes, 1995). In clinical practice, negative countertransferences can be particularly problematic, and theoretically, carry a greater risk of complaints and legal actions stemming from patient's dissatisfaction rather than actual medical errors.

The source of countertransference can be the patient or the clinician (Cabaniss et al., 2017, pp. 254–265); that is, the quality of countertransference is determined by the token of the object relation activated in the patient's or the clinician's mind. To infer the source of countertransference, the clinician should focus on five factors: (a) Could the patient's verbal or non-verbal communication have contributed to the countertransference experienced by the clinician? (b) Is the countertransference similar in quality to the self or object representations included in the object relation dyad activated in the patient's mind? (c) Is the actual physician-patient relationship similar to the relationships established by the patient with other people? (d) Are there any situational factors in the clinician's life that may have contributed to the countertransference?, and (e) Is the actual physician-patient relationship similar to relationships established by the clinician with other people? An affirmative answer to (a), (b), or (c) strengthens the inference that the source of the countertransference is the patient. An affirmative answer to (d) or (e) strengthen the inference that the source of the countertransference is the physician (Tansey & Burke, 1989, pp. 111–132).

If it is established that the source of countertransference is the patient, then countertransference can be classified as concordant or complementary. When a concordant countertransference is experienced, the physician identifies with the self representation that the patient has activated. Therefore, the clinician takes the patient's perspective and empathizes cognitively and affectively with the patient. For example, he or she would think: "If I were in the patient's situation, I would probably believe, feel, and act in a similar way". Then, his or her affects would become similar in quality to those of the patient; therefore, if the patient experiences sadness, the physician would also be able to feel it (Bateman & Holmes, 1995; Mills, 2004). When a complementary countertransference is experienced, the clinician identifies with the object representation that the patient has activated; therefore, the clinician's cognition, affects, and behaviors would be similar to those that the patient perceives and expects from him or her (Bateman, Brown, & Pedder, 2000).

To diagnose the qualities and the kind of countertransference that the physician experiences, he or she must take a pause and reflect: What do I think of this patient? How do I feel when with this patient? How am I behaving with this patient? Let us return to the example of the hypertense patient with poor adherence to drug treatment and suppose that during the interaction with the patient, and prior to the intervention of reduction of the negative transference, the physician asks him or herself the previous questions and realizes that he thinks that the patient is irresponsible for not taking his medications, feels annoyed and critical of him or her, and raises his voice slightly in disgust—this last manifestation corresponds to nonverbal communication. Therefore, the clinician concludes that his or her countertransference is complementary and that he or she is acting exactly like the representation of the critical, and perhaps inconsiderate physician, that the patient has perceived (and probably unconsciously induced) on him or her.

Appropriate management of countertransference in clinical practice, independent of the source of countertransference, includes self-insight, anxiety management, empathy, and conceptualizing ability. Self-insight is the ability to know at a given moment what we feel, think, and how we behave; anxiety management is the ability to control anxiety associated with countertransference so that it does not affects the physician's behavior; empathy is the ability to access the patient's perspective of a given situation; and conceptualizing ability is the ability to make use of a theory (for example, the theory presented herein) to understand the role of the patient in the physician-patient interaction (Hayes, Gelso, & Hummel, 2011). These four techniques overlap and interact with each other, and using them decreases the risk of the clinician acting in response to countertransference.

Self-insight should be used whenever the clinician detects that his or her mood is more intense than what is normal for him or her. When this happens, the physician should take a pause and ask him or herself: What do I think of this patient? How do I feel when I am with this patient? How am I behaving with this patient? This is the most essential factor in managing countertransference.

The management of anxiety associated with countertransference includes the normalization of thoughts and feelings that the clinician experiences towards the patient. To normalize the countertransference experience, it is useful to remember that it is expected and healthy to have feelings and thoughts directed toward patients as people with own personalities (Bateman et al., 2000) and not only as sick people, regardless of whether these feelings and thoughts are pleasant or unpleasant. In addition, the clinician should not take the patient's behavior as something personal against him or herself, but as a behavior that is secondary to the functioning of the patient's mind and to his or her way of relating to others. Upon adhering to this technique, the intensity of anxiety associated with countertransference should decrease considerably.

In the event that the clinician presents a complementary countertransference, that is, when he or she identifies with the object representation included in the dyad activated in the patient's mind, the physician must make a cognitive effort to develop empathy in order to acquire their perspective (Tansey & Burke, 1989, pp. 85–98). For this, it is useful to remember that the patient is also a human being with all the virtues and defects that the physician has as well (Gelso & Hayes, 2007) and should ask him or herself: If I were in the patient's situation how would I feel? What would I think? How would I behave? How would I like to be treated by my clinician? When the physician presents a concordant countertransference, empathy will be implicit, and it will not be necessary to develop it; what he or she should be wary of is over-identification with the patient's self representation, since this may cause the clinician to give the patient just what he or she wants, not what he or she needs. To avoid this, the physician must make an effort to think that in the physician-patient relationship, there must be a certain healthy emotional distance between the physician and the patient and remember that every patient precisely looks for that in a clinician: a professional who will guide them to solve their health problems.

Conceptualizing ability corresponds to the use of psychodynamic psychology to understand the countertransference reactions of clinicians towards their patients (Gelso & Hayes, 2007). After transference and countertransference have been diagnosed, the physician must ask him or herself: How does my countertransference relate to the activated object relation in the patient's mind that dominates the interaction between my patient and me? Is it a complementary or concordant countertransference? Doing so will reduce anxiety and confusion in the physician-patient interaction (Zerbo, Cohen, Bielska, & Caligor, 2013) and will allow the physician to carry out the appropriate technique for transference management.

Finally, there will be unusual occasions when countertransference is so intense that it cannot be handled appropriately, posing a high risk that the clinician would deviate from professional boundaries. Because a good physician-patient relationship is necessary for any successful treatment and the risk of iatrogenesis due to deviation from professional boundaries is high, an uncontrollable countertransference is a rational reason to refer a patient to a colleague. However, before doing this, the physician should try to repair the relationship; if this countertransference has its origin in objective characteristics of the patient, the clinician should discuss this with him or her and give them the opportunity to change. If this last intervention is not effective or if the cause of the countertransference lies in the personal characteristics of the physician, the clinician must apologize for his or her professional limitations, express that the patient deserves to receive better care than he or she could receive with him or her, and finally, offer a referral to another practitioner (Golden & Brennan, 1995; Gunderson & Links, 2014).

## Conclusions

To my knowledge, this is the first paper that presents a psychodynamic framework applicable in clinical practice and accessible to lay clinicians for understanding and managing the physician-patient relationship. Other authors have suggested that this kind of psychodynamic frameworks can be applied in time-constrained clinical settings and even in the absence of a strong and established relationship between the physician and the patient (Zerbo et al., 2013). Theoretically, applying the concepts and techniques outlined in this paper would improve the quality of the physician-patient relationship, and thus improve healthcare outcomes.

Of note, Balint groups were also developed to understand and improve the physician-patient relationship (Yazdankhahfard, Haghani, & Omid, 2019) and nowadays are well-established in different settings and countries (Van Roy, Vanheule, & Inslegers, 2015). These groups are composed by health professionals with different backgrounds—such as general practitioners, nurses, medical specialist, medical residents, and medical students, among others—that discuss psychological aspects of difficult interactions they have had with patients and an expert facilitator helps them understand and manage these psychological aspects from a psychodynamic framework (Van Roy et al., 2015; Yazdankhahfard et al., 2019). Nevertheless, Balint groups provoke anxiety in their participants, and attendees tend to request more theoretical teaching as a way to reduce this anxiety (Graham, Gask, Swift, & Evans, 2009). Perhaps this work could be used to this end as introductory material to the groups.

Clinicians require some level of self-insight and reflective capacity to understand and manage psychodynamic aspects of the physician-patient relationship. Ideally, practitioners should undergo personal psychodynamic psychotherapy to attain these required abilities. In reality, few clinicians can afford personal psychodynamic psychotherapy, but there are more plausible activities that foster self-insight: (a) whenever possible—waiting inside an elevator, taking a shower, laying on the bed, for example—let your mind wonder about recent interpersonal interactions and how you and the other were feeling, thinking and behaving; (b) practice any kind of meditation; and (c) take care of yourself by resting, doing activities for pleasure, and so forth (Gelso & Hayes, 2007).

No one can master a complex phenomenon such as the physician-patient relationship by reading a single paper. I hope this work rises the interest of some health professionals in the psychodynamic understanding and management of the physician-patient relationship, help some of them to put in practice the techniques presented herein, and motivate further reading on the subject. You and your patients could be rewarded.

## Note

1 I refer the reader interested in the history, development, and empirical evidence of psychodynamic concepts to Luborsky and Barret (2006), Ulberg and Dahl (2018) and Yakeley (2018).

## References

[CIT0001] Arbuthnott, A., & Sharpe, D. (2009). The effect of physician–patient collaboration on patient adherence in non-psychiatric medicine. *Patient Education and Counseling*, *77*(1), 60–67. doi:10.1016/j.pec.2009.03.022 19395222

[CIT0002] Auchincloss, E. L. (2015). *The psychoanalytic model of the mind*. Arlington: American Psychiatric Association Publishing.

[CIT0003] Bateman, A., Brown, D., & Pedder, J. (2000). *Introduction to psychotherapy: An outline of psychodynamic principles and practice* (3rd ed.). London: Routledge.

[CIT0004] Bateman, A., & Holmes, J. (1995). *Introduction to psychoanalysis contemporary theory and practice*. London: Routledge.

[CIT0005] Brenner, C. (1974). The drives. In C. Brenner (Ed.), *An elementary textbook of psychoanalysis* (pp. 15–30). New York: Anchor Books.

[CIT0006] Cabaniss, D. L., Cherry, S., Douglas, C. J., & Schwartz, A. (2017). *Psychodynamic psychotherapy: A clinical manual* (2nd ed.). Chichester: John Wiley & Sons.

[CIT0007] Caligor, E., Kernberg, O. F., & Clarkin, J. F. (2007). *Handbook of dynamic psychotherapy for higher level personality pathology*. Arlington: American Psychiatric Publishing.

[CIT0008] Caligor, E., Kernberg, O. F., Clarkin, J. F., & Yeomans, F. E. (2018). *Psychodynamic therapy for personality pathology treating self and interpersonal functioning*. Washington: American Psychiatric Association Publishing.

[CIT0009] Carter, M., & Shieh, J. (2015). *Guide to research techniques in neuroscience* (2nd ed.). San Diego: Academic Press.

[CIT0010] Clarkin, J. F., & Yeomans, F. (2013). Managing negative reactions to clients with borderline personality disorder in transference-focused psychotherapy. In A. W. Wolf, M. R. Goldfried, & J. C. Muran (Eds.), *Transforming negative reactions to clients: From frustration to compassion* (pp. 175–188). Washington: American Psychological Association.

[CIT0011] Eagle, M. N. (2012). Theories of motivation. In G. O. Gabbard, B. E. Litowitz, & P. Williams (Eds.), *Textbook of psychoanalysis* (2nd ed., pp. 39–52). Arlington: American Psychiatric Publishing.

[CIT0012] Gabbard, G. O. (2014). *Psychodynamic psychiatry in clinical practice* (5th ed.). Arlington: American Psychiatric Publishing.

[CIT0013] Gelso, C. J., & Hayes, J. A. (2007). *Countertransference and the therapist’s inner experience: Perils and possibilities*. New Jersey: Lawrence Erlbaum Associates.

[CIT0014] Gilmore, K. J., & Meersand, P. (2014). *Normal child and adolescent development: A psychodynamic primer*. Arlington: American Psychiatric Publishing.

[CIT0015] Golden, G. A., & Brennan, M. (1995). Managing erotic feelings in the physician-patient relationship. *Canadian Medical Association Journal*, *153*(9), 1241–1245. 7497386 PMC1487496

[CIT0016] Gómez, G., & Aillach, E. (2013). Ways to improve the patient–physician relationship. *Current Opinion in Psychiatry*, *26*(5), 453–457. doi:10.1097/YCO.0b013e328363be50 23867657

[CIT0017] Graham, S., Gask, L., Swift, G., & Evans, M. (2009). Balint-style case discussion groups in psychiatric training: An evaluation. *Academic Psychiatry*, *33*(3), 198–203. doi:10.1176/appi.ap.33.3.198 19574515

[CIT0018] Gunderson, J. G., & Links, P. (2014). *Handbook of good psychiatric management for borderline personality disorder*. Washington: American Psychiatric Publishing.

[CIT0019] Hall, A. J., Horgan, T. G., & Murphy, N. A. (2019). Nonverbal communication. *Annual Review of Psychology*, *70*, 270–294. doi:10.1146/annurev-psych-010418-103145 30256720

[CIT0020] Haskard Zolnierek, K. B., & DiMatteo, R. (2009). Physician communication and patient adherence to treatment: A meta-analysis. *Medical Care*, *47*(8), 826–834. doi:10.1097/MLR.0b013e31819a5acc 19584762 PMC2728700

[CIT0021] Hayes, J. A., Gelso, C. J., & Hummel, A. M. (2011). Managing countertransference. *Psychotherapy*, *48*(1), 88–97. doi:10.1037/a0022182 21401279

[CIT0022] Hersh, R. G., Caligor, E., & Yeomans, F. E. (2016). *Fundamentals of transference-focused psychotherapy applications in psychiatric and medical settings*. Switzerland: Springer.

[CIT0023] Hilgard, E. R. (1980). The trilogy of mind: Cognition, affection, and conation. *Journal of the History of the Behavioral Sciences*, *16*(2), 107–117. doi:10.1002/1520-6696(198004)16:2<107::aid-jhbs2300160202>3.0.co;2-y 11608381

[CIT0024] Hofsess, C. D., & Tracey, J. G. (2010). Countertransference a prototype: The development of a measure. *Journal of Counseling Psychology*, *57*, 52–67. doi:10.1037/a0018111 21133560

[CIT0025] Jacobson, E. (1964). *The self and the object world*. New York: International Universities Press.

[CIT0026] Jani, B. D., Blane, D. N., & Mercer, S. W. (2012). The role of empathy in therapy and the physician-patient relationship. *Forschende Komplementarmedizin (2006)*, *19*(5), 252–257. doi:10.1159/000342998 23128100

[CIT0027] Joseph, B. (1985). Transference: The total situation. *The International Journal of Psychoanalysis*, *66*(4), 447–454.

[CIT0028] Kelley, J. M., Kraft-Todd, G., Schapira, L., Kossowsky, J., & Riess, H. (2014). The influence of the patient-clinician relationship on healthcare outcomes: A systematic review and meta-analysis of randomized controlled trials. *PLoS One*, *9*(4), e94207. doi:10.1371/journal.pone.0094207 24718585 PMC3981763

[CIT0029] Kernberg, O. F. (2018). A new formulation of supportive psychodynamic psychotherapy. In O. Kernberg (Ed.), *Treatment of severe personality disorders: Resolution of aggression and recovery of eroticism* (pp. 131–149). Washington: American Psychiatric Association Publishing.

[CIT0030] Luborsky, L., & Barret, M. S. (2006). The history and empirical status of key psychoanalytic concepts. *Annual Review of Clinical Psychology*, *2*(1), 1–19. doi:10.1146/annurev.clinpsy.2.022305.095328 17716062

[CIT0031] Lussier, M.-T., & Richard, C. (2005). Doctor-patient communication: Complaints and legal actions. *Canadian Family Physician*, *51*(1), 37–39. 15732220 PMC1479583

[CIT0032] Mast, M. S. (2007). On the importance of nonverbal communication in the physician–patient interaction. *Patient Education and Counseling*, *67*(3), 315–318. doi:10.1016/j.pec.2007.03.005 17478072

[CIT0033] Mills, J. (2004). Countertransference revisited. *The Psychoanalytic Review*, *91*(4), 467–515. doi:10.1521/prev.91.4.467.48751 15491945

[CIT0034] Pitt, D. (2018). Mental Representation, Zalta, E.N. (Ed.) *The Stanford Encyclopedia of Philosophy*. Retrieved from https://plato.stanford.edu/archives/win2018/en.

[CIT0035] Riedl, D., & Schüssler, G. (2017). The influence of doctor-patient communication on health outcomes: A systematic review. *Zeitschrift für Psychosomatische Medizin und Psychotherapie*, *63*(2), 131–150. doi:10.13109/zptm.2017.63.2.131 28585507

[CIT0036] Russell, B. (2016). *Fundamentos de filosofía* [An outline of philosophy]. D. F.: Debolsillo.

[CIT0037] Segarra, R., & Eguíluz, I. (2013). Afectividad. In L. Eguíluz & R. Segarra (Eds.), *Introducción a la psicopatología Una visión actualizada* [Introduction to Psychopathology An Updated Overview] (3rd ed., pp. 115–160). Madrid: Editorial Médica Panamericana.

[CIT0038] Squier, R. W. (1990). A model of empathic understanding and adherence to treatment regimens in practitioner-patient relationships. *Social Science & Medicine*, *30*(3), 325–339. doi:10.1016/0277-9536(90)90188-x 2408150

[CIT0039] Tansey, M. J., & Burke, W. F. (1989). *Understanding countertransference: From projective identification to empathy*. New Jersey: The Analytic Press.

[CIT0040] Ulberg, R., & Dahl, H. J. (2018). Empirical support for the psychoanalytic concepts. *The Lancet. Psychiatry*, *5*(7), 543–544. doi:10.1016/S2215-0366(18)30208-6 29941134

[CIT0041] Van Roy, K., Vanheule, S., & Inslegers, R. (2015). Reserach on Balint groups: A literature review. *Patient Education and Counseling*, *98*(6), 685–694. doi:10.1016/j.pec.2015.01.014 25681874

[CIT0042] Yakeley, J. (2018). Psychoanalysis in modern mental health practice. *The Lancet. Psychiatry*, *5*(5), 443–450. doi:10.1016/S2215-0366(18)30052-X 29574047

[CIT0043] Yazdankhahfard, M., Haghani, F., & Omid, A. (2019). The Balint group and its application in medical education: A systematic review. *Journal of Education and Health Promotion*, *8*, 124. doi:10.4103/jehp.jehp_423_18 31334276 PMC6615135

[CIT0044] Yeomans, F. R., Clarkin, J. F., & Kernberg, O. F. (2015). *Transference-focused psychotherapy for borderline personality disorder: A clinical guide*. Arlington: American Psychiatric Publishing.

[CIT0045] Zerbo, E., Cohen, S., Bielska, W., & Caligor, E. (2013). Transference-focused psychotherapy in the general psychiatry residency: A useful and applicable model for residents in acute clinical settings. *Psychodynamic Psychiatry*, *41*(1), 163–181. doi:10.1521/pdps.2013.41.1.163 23480166

